# Low-Cost Monitoring of Spatiotemporal Changes in Forest Cover: Implications for Conservation in a Case in Quindío (Colombia)

**DOI:** 10.1007/s00267-026-02491-4

**Published:** 2026-07-17

**Authors:** María Eunice Quintero-Gallego, Alexander Ariza, José Joaquín Vila-Ortega, Mauricio Quintero-Ángel, Gloria Yaneth Florez-Yepes

**Affiliations:** 1 Universidad Católica de Manizales, (Master’s program in Remote Sensing), Manizales (Caldas), Colombia; 2 Universidad Politécnica de Madrid (Geo-Qubidy Research Group), Madrid, Spain; 3 Universidad del Quindío, (Faculty of Engineering), Armenia, (Quindío), Colombia; 4 Universidad del Valle, seccional Palmira, Palmira, Colombia

**Keywords:** Remote detection, Spectral indices, Google Earth engine, Landscape fragmentation, Conservation

## Abstract

Globally, changes in forest cover and fragmentation are a conservation concern. Updated information on their state is required, but it is often costly and difficult to obtain. This study evaluated the feasibility of using low-cost technologies, such as freely available remote sensing data and software, to monitor spatiotemporal changes in forest cover and their relationship to landscape dynamics in terms of structure, shape, and configuration. The latter, using a tropical ecosystem in Quindío (Colombia), between 2002 and 2019, as an example. This is particularly relevant given the complexities of land cover classification in tropical ecosystems and the presence of red howler monkeys (*Alouatta seniculus*), whose populations are threatened by changes in landscape dynamics. Satellite imagery (Landsat 7, Landsat 8, and Sentinel-2) and a 30-m Digital Elevation Model were processed using analysis algorithms: CART, Random Forest, Naive Bayes, and Support Vector Machine using six spectral indices (NDVI;EVI;SAVI;CIgreen;NDBI;NDWI). The classifications with the highest Kappa index for the Fragstats analysis were CART for 2002 under spectral bands with topographic variables (C4), and Random Forest for 2014 and 2019 under spectral indices with topographic variables (C5). The combination of spectral indices and topographic variables (C5) with Random Forest achieved the highest performance, with Kappa values up to 0.882 and detection accuracy above 95%. The latter, indicating superior capability for detecting land cover changes. Landscape metrics were also calculated using six indicators (NP,LPI, AREA_MN,SHAPE_MN,FRAC_MN,ENN_MN). Between 2002 and 2019, forest cover decreased by 4.05%, from 77.42% to 73.37%, reflecting quantifiable landscape transformation during the study period. The results demonstrate that open-source remote sensing data and software are useful and reliable to study land cover dynamics, while reducing processing costs, time, and resource demands. This cost-efficiency is particularly relevant in the Global South, where technological and budgetary constraints often limit effective monitoring of landscape change and informed conservation decision-making.

## Introduction

The scientific community recognizes that humans have put a significant pressure on the biosphere (Steffen et al. 2018), to the extent that it is suggested that the Earth system is experiencing a sixth mass extinction driven by humans (Ceballos and Ehrlich 2023). Current extinction rates of animal species are hundreds or thousands of times higher than the background rates that existed before the Agricultural Revolution (Ceballos et al. 2020; Pimm et al. 2014). In light of the current planetary crisis, to prevent biodiversity decline and species extinction, governments worldwide have introduced laws and policies to protect endangered species and their habitats, restrict destructive human behaviors, and mitigate rising extinction rates (Brockett et al. 2023; Evans and Malcom 2021). Granting legal protection to an endangered species or its habitat is considered a major milestone for conserving nature and halting the loss of biodiversity (Geldmann et al. 2019; Shirey et al. 2025). However, human pressures have increased within protected areas, with the most significant changes observed in the tropics (Geldmann et al. 2019). Fragmentation and biodiversity loss continue around the world, given that legal obligations may be ineffective if the rule of law is not properly enforced (Geldmann et al. 2013, 2019; Li et al. 2024; López-Bao et al. 2015).

In conservation, monitoring is recognized as essential for quantifying population trends, documenting ecosystem conditions, and determining the effectiveness of management interventions (Lindenmayer et al. 2020). Therefore, monitoring and evaluation are essential for ensuring the effectiveness of international biodiversity frameworks (Brockett et al. 2023). It is recognized that flexible, efficient, and effective monitoring and enforcement methods are essential for conservation policies realize their full benefit (Evans and Malcom 2021; Lahoz-Monfort and Magrath 2021). However, the impacts of conservation measures on habitat loss may not be immediately apparent (Li et al. 2024) and due to the rapid rate of biodiversity loss and limited funding, conservation programs must decide which conservation actions to prioritize (Buxton et al. 2020). Consequently, despite their importance, many threatened species and habitats worldwide remain unmonitored or poorly monitored (Lindenmayer et al. 2020; Pickens et al. 2025; Senior et al. 2024).

Inefficient monitoring undermines imperiled species conservation, by limiting decision-making with information based on the status of species’ habitats or the habitat protection actions that may go unenforced (Kéry and Schmidt 2008). Similarly, a lack of monitoring and evidence regarding the effectiveness and return on investment of conservation actions can create the perception that these actions are ineffective or poor value (Lindenmayer et al. 2020). Also, the lack of regular monitoring and enforcement sends a message to other actors that they may likely destroy habitats without facing consequences (Evans and Malcom 2021). Therefore, in order to conserve ecosystems, it is important to monitor changes in land cover and use, and to have updated, and reliable information about these changes and their long-term replacement rates and degrees (Dinssa et al. 2016; Kennedy et al. 2014; Pickens et al. 2025). This information is necessary for making decisions regarding territorial planning, policies, investments, sustainable development, and management (Chernenkova et al. 2023).

Despite the increased availability of remote sensing (RS) data in recent years, it remains underused for systematic conservation monitoring. This is partly due to a lack of simple tools and concerns about relevance, resolution, accessibility, and processing (Evans & Malcom, 2021; Pettorelli and Schulte to Bühne 2023; Skidmore et al. 2015). The tools that allow users to explore, analyze, and process continuous change detection and classification algorithm outputs in a simplified way are still missing (Arévalo et al. 2020). Likewise, RS has traditionally been a complex and expensive process for conservation. RS demands specialized paid software; high-capacity computers; and highly trained personnel. Also, acquiring paid images is usually required. This has limited its use in supporting conservation decisions, particularly in middle and low-income countries, which are mostly located in areas with high biodiversity (Ayyamperumal et al. 2024; Pettorelli et al. 2025; Velastegui-Montoya et al. 2023; Vijayakumar et al. 2024). These countries face many development challenges, so environmental investments are not usually a priority. This results in underfunded government agencies, inefficient monitoring and unenforced habitat protection actions. Therefore, it is required to explore the use of technologies and methodologies that facilitate obtaining results with high degree of optimization of processing time and resources, such as those provided at no cost for noncommercial and research use by the Google Earth Engine (GEE) platform.

GEE permits processing and analyzing information in the cloud, through an extensive catalog of petabytes of satellite images from different sensors and the use of machine learning algorithms configured with the JavaScript and Python programming languages (Tamiminia et al. 2020; Tsai et al. 2018). Thus, the classification process of wildland covers can be semi-automated, consuming less time and resources to provide information that meets the needs of different stakeholders. This information is essential for making timely and reliable conservation decisions and allows for continuous low-cost monitoring. However, there is a need to expand knowledge using GEE in middle and low-income countries, particularly in environmental and conservation monitoring (Velastegui-Montoya et al. 2023; Vijayakumar et al. 2024). Large-scale monitoring programs to efficiently and automatically detect disturbances to wildlife habitat are needed, especially in hot spots of biodiversity in middle and low-income countries (Alencar et al. 2020; Evans & Malcom, 2021; Pickens et al. 2025). In conservation, it is estimated that the continued improvements to automated change detection methods and their adoption by regulatory authorities have the potential to close a significant gap in biodiversity protection (Evans and Malcom 2021; Pettorelli et al. 2025).

In this context, the aim of this paper was to evaluate the potential of using low-cost technologies, such as freely available remote sensing data and software, to monitor spatiotemporal changes in forest cover and their relationship with landscape dynamics in function of its structure, shape, and configuration. The latter, taking a tropical ecosystem in Quindío (Colombia), between 2002 and 2019, as an example. This example is relevant because land cover and use classifications present complexities in tropical ecosystems, such as high species biodiversity, prevalent cloud cover and swift landscape shifts at a fine-grained level, which undermine the efficacy of conventional remote sensing techniques (Anaya et al. 2023; Kang et al. 2020). In terms of conservation, the example is also relevant given the presence of two nature reserves, where the survival of red howler monkey (*Alouatta seniculus*) populations is threatened by changes in landscape dynamics (Quintero-Gallego et al. 2018). Howler monkeys are among the last diurnal large mammals found in the Andean region in Colombia, so protecting them as an umbrella species could be an important conservation tool for the remaining Andean forests and their biodiversity (M. C. Gómez-Posada 2014).

## Materials And Methods

### Study Area

**Fig. 1:**
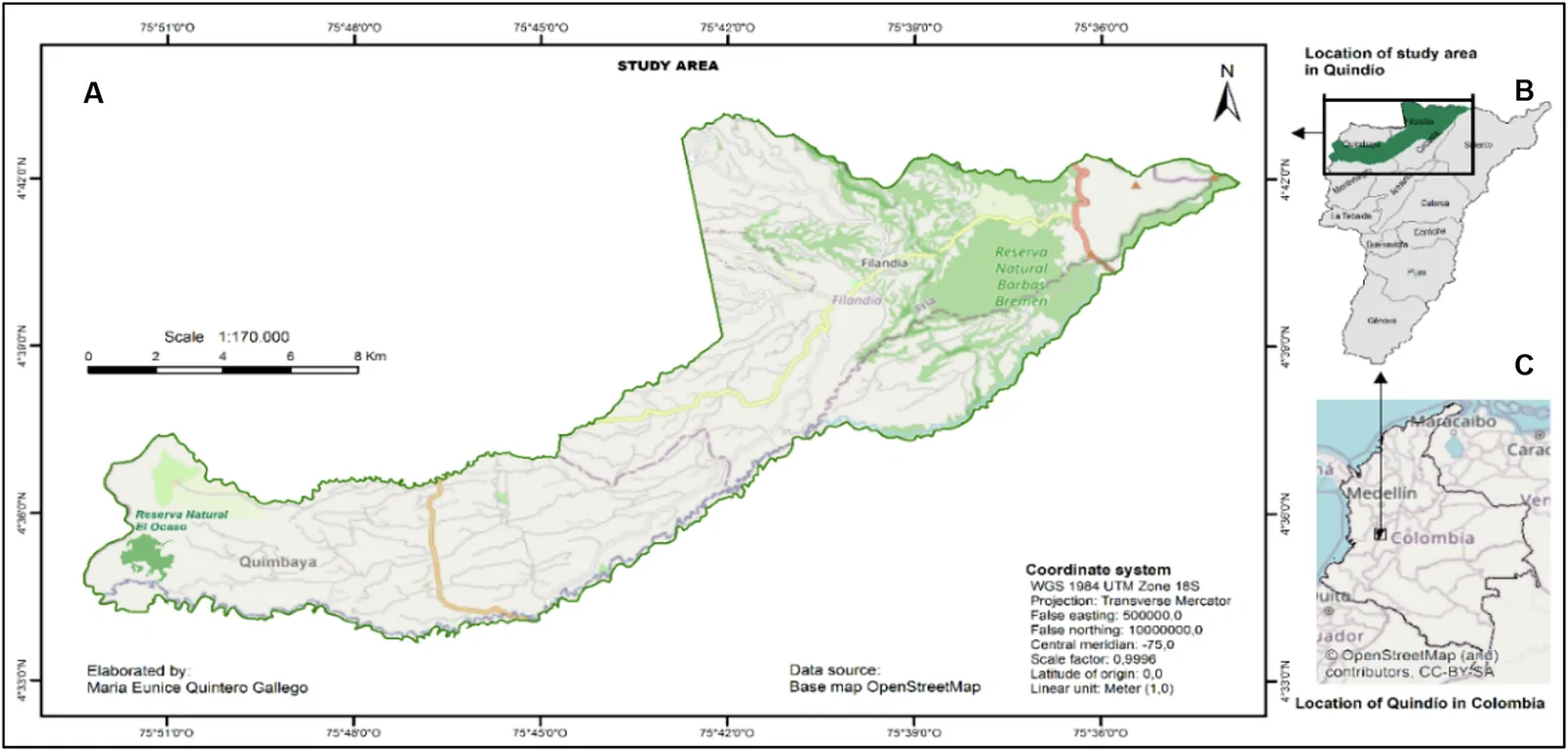
**A** Location of the study area between Quimbaya and Filandia; **B** Location of the study area in the Quindío region; **C** Location of the Quindío region in Colombia in the northern part of South America

The study area covers 20,098 ha of land in the Quindío region (Colombia), between the municipalities of Filandia and Quimbaya. The area has an altitude ranging from 975 to 2005 m, a mean annual temperature between 17 and 24 °C, a relative humidity between 76% and 87%, and an annual precipitation ranging from 1691 to 2890 mm. The precipitation is distributed over two major rainy seasons, from October to December and from April to May and two less rainy season or dry seasons, from January to March and from June to September (Gómez-Hoyos et al. 2014; Valencia 2017). The area includes formations of premontane moist forest (PM-mf) and premontane very moist forest (PM-vmf) (Agudelo and Vélez 2001) (Fig. 1). The region encompasses two areas of interest, where conservation processes of various flora and fauna species are underway. The first is La Montaña del Ocaso Natural Reserve, which covers 148 hectares and has over 350 species of flora, more than 100 species of birds, and several species of mammals, including the red howler monkey, the two-toed sloth (*Choloepus hoffmanni*), and the white-faced capuchin (*C. capucinus*), as well as diverse insect species (Agudelo and Vélez, 2001). The second area corresponds to the Bremen-La Popa Natural Reserve in the Barbas-Bremen Soil Conservation District. The reserve contains forests and wetlands with various flora and fauna. Some species are endemic or have limited distributions, while others are threatened. The area is also considered important for bird conservation (CRQ and FCA 2014, Gómez-Hoyos et al. 2014). It also provides goods and ecosystem services, such as water for human populations and crop irrigation (Valencia 2017; Gómez-Hoyos et al. 2014).

### Methodology

**Fig. 2:**
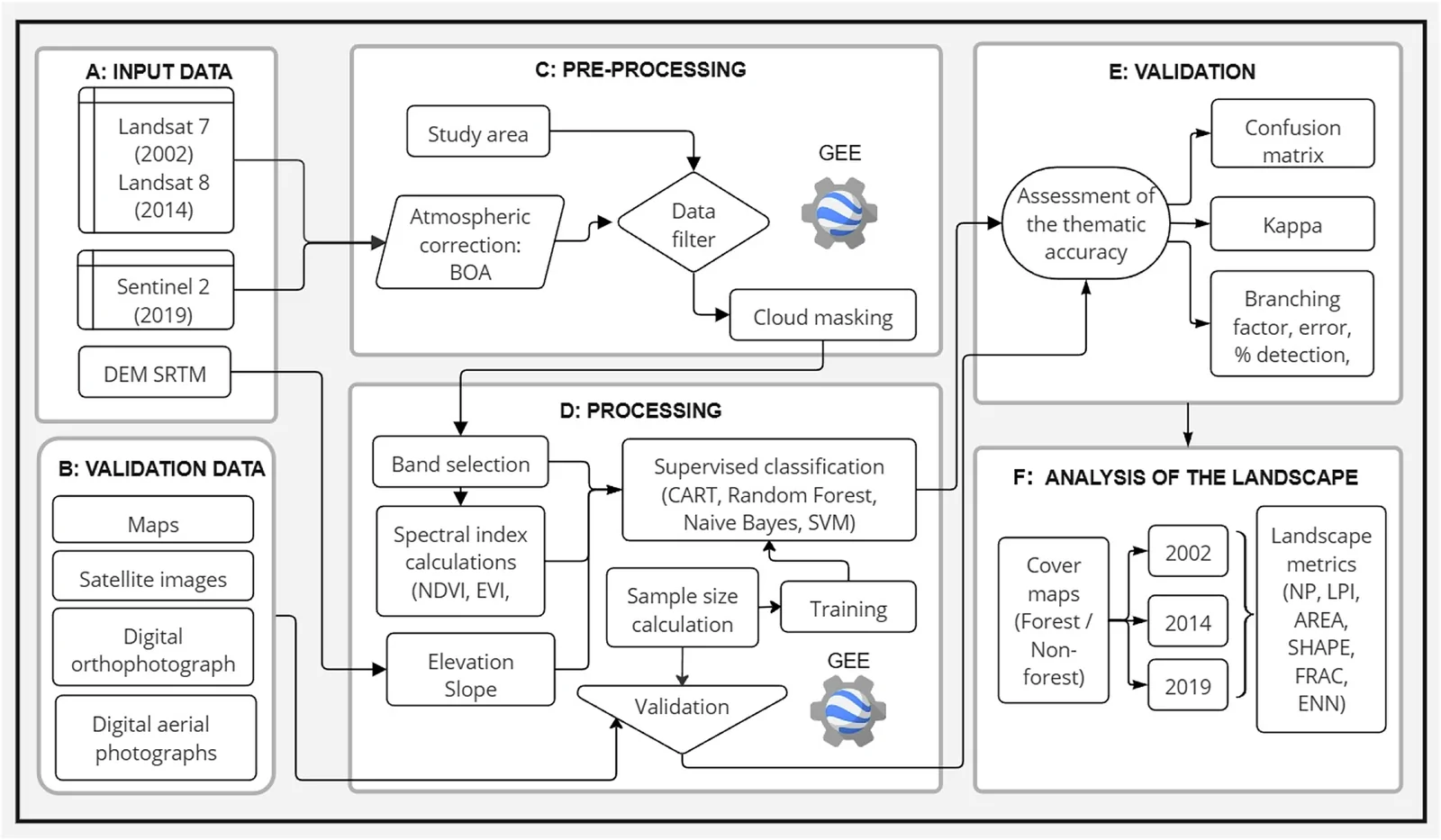
Diagram of the methodological flow used in the study. Section A shows the input data used for classification (see supplementary material 1 for details). Section B shows the data used for validation (see Table 3 for details). Section C shows the pre-processing stage, and section D shows the data processing stage. Section E shows the validation process, and section F shows the landscape analysis

The methodological process was divided into five general phases: (i) Data input, (ii) Pre-processing, (iii) Processing, (iv) Validation, and (v) Analysis of the landscape’s changes in composition, shape, and configuration, described ahead (Fig. 2).

i. Data input: For this 17-year study period, three satellite images were selected, corresponding to the years 2002, 2014, and 2019. The selection criteria prioritized images with low cloud cover and temporal intervals that permitted effective spatial and temporal analysis for conservation decision-making purposes (Supplementary material 1). Although the study area is located within a tropical convergence zone, which usually results in higher cloudiness, the selected images had cloud covers mostly below 10%. Notably, the area of interest remained largely unaffected by clouds, ensuring the quality and reliability of the data for analysis.
ii. Pre-processing: The preprocessing steps were performed separately for Landsat and Sentinel data. This accounts for data quality and comparability throughout the study period.

- Landsat data (2002 and 2014): Collections of surface reflectance (or bottom-of-atmosphere, BOA) were extracted based on date, percent cloud cover, and the boundaries of the study area. In the case of cloud cover, any percentage below 10% was considered for extraction. The reason behind the 10% threshold was to balance image quality with available data within the analyzed period to better assess land cover change. For the 2014 image, the cloud masking procedure of the QA_PIXEL band was utilized to provide a pixel level quality assessment of cloudy pixels (USGS 2021). This VEGA cloud mask provides pixel assessment information for removing cloud affected pixels to limit misclassification during analysis. A pansharpening fusion technique was applied to the 2002 and 2014 Landsat images to improve their spatial resolution and visual interpretability for selecting training data.
- Sentinel-2 data (2019): Surface reflectance products were extracted with less than 5% of cloud cover to prioritize the highest available quality. The area of interest was designated (clipped) using the study zone boundary. The area of interest, the image extent, and the other usable imagery were not affected by the clip. No additional cloud mask was applied based on the low cloud coverage in the usable Sentinel-2 scene selection.

(iii) Processing: from images with surface reflectance, six spectral indices were calculated: NDVI “Normalized Difference Vegetation Index” (Rouse Jr et al. 1974); EVI “Enhanced Vegetation Index” (Huete et al. 2002); SAVI “Soil Adjusted Vegetation Index” (Huete 1988); CIgreen “Chlorophyll Index Green” (Gitelson et al. 2003); NDBI “Normalized Difference Built-up Index” (Zha et al. 2003); and NDWI “Normalized Difference Water Index” (Gao, 1996) (See supplementary material 2 for formulas and examples of their use in RS). These spectral indices were added as individual bands for the classification process; likewise, the elevation and slope variables were extracted from the DEM.

These aggregate data were used as auxiliary data for analysis, grouped into six combinations corresponding to:

- Combination 1 (C1): Spectral bands
- Combination 2 (C2): Spectral indices
- Combination 3 (C3): Spectral bands + spectral indices
- Combination 4 (C4): Spectral bands + topographic variables
- Combination 5 (C5): Spectral indices + topographic variables
- Combination 6 (C6): Spectral bands + spectral indices + topographic variables

Subsequently, the statistical sample of training points was calculated according to the number of pixels in the study area (223,319) with 95% confidence and 5% margin of error, yielding 384 points. These points were located in the study area in simple random fashion, using the ArcGIS 10.6 (ESRI-Environmental Systems Research Institute., 2017) random points tool^1^, considering two criteria: (i) a distance of 100 m from the edge of the study area; and (ii) a distance of 200 m between each point. Training was conducted using these points to identify forest and non-forest areas in each of the three images, based on the physiognomy, patterns, and tonality of the observed covers. Next, four machine learning algorithms were analyzed with the six combinations resulting from the spectral bands, spectral indices, and elevation and slope topographic variables. The algorithms corresponded to Classification and Regression Tree (CART) (Breiman et al. 1984), Naive Bayes (Bayes 1763), Support Vector Machines (SVM) (Cortes and Vapnik 1995), and Random Forest (RF) (Breiman, 2001). The Random Forest algorithm was configured with 500 decision trees because errors stabilize before reaching this number of classification trees (Belgiu and Drăgut 2016; Hermosilla et al. 2022) (See Table 1 for descriptions of the algorithms and examples of their use in RS).

**Table 1:** Algorithms used to perform the supervised classification

Algorithm	Description	Examples of use in remote sensing
Classification and Regression Tree (CART) (Breiman et al. 1984)	This algorithm applies a multi-stage binary decision-making system to classify images. In each stage, the pixels are divided according to the binary classification rule. The groups of pixels can be divided further in function of the tree pruning and growth parameters, until achieving optimal classification. These can be sensitive to small changes in data and training parameters (Tsai et al. 2018 and Sluiter & Pebesma, 2010).	Classify images using remote sensing data (B. H. Yang and Li 2013; C.-C. Yang et al. 2003); Map the percent tree cover (Cilek et al. 2022); Estimate the impact of land use and cover changes on evapotranspiration (Pande et al. 2024); Classify land use and land cover (Bogale et al. 2025; Zhao et al. 2024).
Naive Bayes (Bayes, 1763)	It is used to calculate conditional probabilities, understood as the probability of an event occurring given that another event has already occurred. It is based on the conditional probability theorem or also called Bayes’ Theorem, which assumes that each characteristic makes an independent contribution equal to the result (Webb et al. 2010; F. Parra, 2019).	Classify land covers using remote sensing data (Gupta et al. 2025; Iqbal et al. 2021); Advanced landslide detection (Al-Batah et al. 2024); Aquatic ecosystems monitoring (Cui et al. 2025)
Support Vector Machines (SVM) (Cortes & Vapnik, 1995)	The SVM focuses exclusively on the training samples (support vectors) that are closest in the space of characteristics of the optimal limit between the classes (Maxwell et al. 2018). The method incorporates an algorithm to find the optimal limit, which maximizes the separation between the support vectors, minimizing classification error. In case of two separable classes, the method adjusts a hyperplane in the middle of a space with maximum margin. For overlapping classes, the method minimizes the distance to the margin of incorrectly classified points (Sluiter & Pebesma, 2010).	Classify images using remote sensing data (Xiong et al. 2010); Aquatic ecosystems monitoring (Cui et al. 2025); Advanced landslide detection (Al-Batah et al. 2024); Forage monitoring and biomass prediction(Zwick et al. 2024); Classify land use and land cover (Bogale et al. 2025; Zhao et al. 2024).
Random Forest (RF) (Breiman, 2001)	This algorithm is a mix of individual decision trees, where each one depends on the values of a random vector sampled apart from the input vector. The prediction for each new observation is obtained when adding the predictions of all the trees that compose the model and each tree casts a single vote and, finally, the decision with the highest number of votes is the algorithm’s prediction (Breiman, 2001; Sluiter & Pebesma, 2010 and Maxwell et al. 2018).	Remote sensing image classification (Pazhanikumar & KuzhalVoiMozhi, 2024): Aquatic ecosystems monitoring (Cui et al. 2025); Restoration Change Monitoring (Brown et al. 2024); Characterization of tropical forests (Singhal et al. 2024); Advanced landslide detection (Al-Batah et al. 2024); Classify land use and land cover (Bogale et al. 2025; Zhao et al. 2024).

(iv) Validation: To evaluate and quantify the accuracy of the supervised classification, a simple random sampling approach was applied to the two target classes (Forest / Non-Forest), following the accuracy assessment principles of Congalton and Green (2019) and (Olofsson et al. 2014). The sample size (n) was determined using the finite population correction formula (Eq. 1).

1 $$\,{\boldsymbol{n}}=\frac{{\boldsymbol{N}}* {{\boldsymbol{Z}}}^{{\bf{2}}}* {\boldsymbol{p}}* {\boldsymbol{q}}}{{{\boldsymbol{e}}}^{{\bf{2}}}* \left({\boldsymbol{N}}-{\bf{1}}\right)+\,{{\boldsymbol{Z}}}^{{\bf{2}}}* {\boldsymbol{p}}* {\boldsymbol{q}}}$$

Where,

*N* is the total number of pixels in the study area (232,307);

*Z* is the 95% confidence level (1.96);

*p* and *q* are the expected and complementary probabilities, both of which are set to 0.5 to achieve maximum variability.

*e* is the specified error, set to 0.05.

A total of 384 points were assigned to the sample based on a Pareto distribution of 70% to training and 30% to validation for each class and year. This approach promoted balance between the forest and non-forest categories. Table 2 summarizes the training and validation datasets for each study period.

**Table 2:** Composition of the training and validation datasets for each land cover class by year

Year	Class	Training points	Validation points	Total points	% of total
2002	Forest	134	58	192	50%
	Non-Forest	134	58	192	50%
2014	Forest	134	58	192	50%
	Non-Forest	134	58	192	50%
2019	Forest	134	58	192	50%
	Non-Forest	134	58	192	50%

The validation reference data included very high spatial resolution imagery from Google Earth, PlanetScope (NICFI collection, Norway’s International Climate and Forests Initiative Satellite Data Program), official land use and cover maps, and Forest-Non-Forest thematic maps from the Institute of Hydrology, Meteorology and Environmental Studies (IDEAM, for the term in Spanish), as well as aerial photos from the Agustín Codazzi Geographic Institute (IGAC, for the term in Spanish) and SIG Quindío (Table 3). Confusion matrices and Cohen’s Kappa coefficient were used in the GEE to assess classification accuracy.

**Table 3:** Data input used for validation

Element	Spatial resolution /Scale	Year	Source
“Land Cover Map. Adaptation Corine Land Cover. Republic of Colombia”	30 m / 1:100.000	2002	IDEAM https://www.colombiaenmapas.gov.co/?e=-76.04892929265479,4.028702460820694,-75.23044784734267,4.768090398395542,4686&b=igac&u=63&t=202&servicio=878.
Collection: “Norway’s International Climate and Forests Initiative Satellite Data Program (NICFI)” Level 1	5 m	2019	Planet Scope Tropical Normalized Analytical Biannual File Mosaic of base map NICFI_Level_1 Identification: 654348 https://www.planet.com/explorer/
Satellite images	15 m	2002 2014 2019	“Google Earth Pro http://www.google.com/intl/es/earth/index.html”
Forest layer – no forest SBQ_SMBYC_BQNBQ_V6.tif	30 m / 1:100.000	2000 2014	“IDEAM Forest and carbon monitoring system SMBYC http://smbyc.ideam.gov.co/MonitoreoBC-WEB/reg/indexLogOn.jsp”
Digital orthophotography	5 m / 1:10.000	2018–2019	“S.I.G. Quindío http://200.21.93.53/sigquindioii/VisorGeneral.aspx”
Digital aerial photographs	14 m / 1:28.000	2003	IGAC Flight C-2699, photographs Nos. 0125, 0170 and 0171

From the confusion matrix, the overall accuracy (number of correctly classified points divided by the total), omission error (reference samples not picked up by the classification; 1—producer’s accuracy), commission error (the number of pixels that are assigned to a particular category that do not correspond to reality; 1 - user’s accuracy), producer’s accuracy (probability that a reference pixel is classified correctly), and user’s accuracy (probability that a classified pixel correctly represents its class on the ground) (Banko 1998; Chuvieco, 2007). Cohen’s kappa coefficient (Chuvieco 1998) was used to evaluate the consistency between the classification and reference data. This could be rated as high ( > 0.75), good (0.40–0.75), or poor ( < 0.40) correspondence (Grauwer and Argipova 2014).

To provide an accurate assessment of the model, the external layers were used only as supplementary reference inputs and not as substitutes for the reference layers that created confusion matrices and the Kappa index based on an independent sample of 384 samples. Referential data sources with higher resolution, such as the Google Earth platform and the Planet NICFI collection, were used wherever possible to strengthen visual validation. As mentioned above in Table 3, in 2002 it was necessary to use only the official thematic maps completed at a scale of 1:100,000 with a spatial error of less than one pixel ( < 30 m). This spatial error is equivalent to Landsat 7 ETM+ imagery with a resolution of 30 m, which is sufficient for validation classification (Table 4).

**Table 4:** Indices used to analyze changes in the landscape structure and configuration

Category	Indices	Level applied	Description
**Composition**	Number of Patches (NP)	Class and landscape	This index quantifies the number of patches of a particular class and provides an estimate of the degree of fragmentation and spatial complexity of the structure of an ecosystem (cover type). The index ranges from 1 to infinity; the higher the value, the greater the degree of fragmentation.
	Largest patch index (LPI)	Class	It measures the largest patch of a given class as a percentage of the total landscape and is used to indicate relative size. It is equivalent to the area (m²) of the particular patch divided by the total area of the landscape, multiplied by 100. The value ranges from zero (0) to one hundred (100) and approaches zero when the largest patch of a corresponding type is small and approaches one hundred if the patch is large. It is a measure of dominance and shows high values in areas with greater coverage and occupation or representativeness in the landscape.
	Mean size of the Patches (AREA_MN)	Class	This index “is equal to the sum of the areas (m2) of all patches corresponding to a specific ecosystem, divided by the number of patches of that type” (McGarigal et al. 2012). It ranges from 0 to infinity.
**Shape**	Average Shape Index of patches (SHAPE_MN)	Class and landscape	This index calculates the complexity of patch shape compared to a standard shape. This index calculates the complexity of a patch’s shape compared to a standard shape. It is equal to the sum of all landscape edges and edge segments that envelop a given patch type, divided by the square root of the total landscape area (m²), adjusted by a standard constant for circular (vector) or square (raster) landscapes. This index ranges from 1 to infinity. When the value is close to 1, the patch is circular. As the patch becomes more irregular and/or the length of its edges increases, the value increases without limit.
	Mean fractal dimension (FRAC_MN)	Class and landscape	It measures the complexity of each fragment. Values range from 1 to 2, with values close to 1 indicating relatively simple shapes (such as circular or square) and values close to 2 indicating more complex shapes.
**Configuration**	Average distance to the nearest neighbor (ENN_MN)	Class	This index measures the distance between a patch and its nearest neighboring patch of the same class. The result shows the degree to which patches of the same class or cover are continuous or isolated.

Finally, the results of the confusion matrix are expressed in terms of four accuracy measurements: (i) Branching factor (BF): Indicates the rate of mislabeled cover points (Eq. 2); (ii) Error factor (EF): indicates the rate of omitted cover points (Eq. 3); (iii) Detection percentage (%D): indicates the covers correctly identified by the proposed methodology (Eq. 4); and (iv) Quality percentage (%Q): indicates the probability that a cover identified by the classifiers is true (Eq. 5) (Lee et al. 2003).

2 $${Branching}\,{factor}\left({BF}\right)=\frac{{False}\,{positives}}{{true}\,{positives}}$$

3 $${Error}\,{factor}({EF})=\frac{{False}\,{negatives}}{{true}\,{positives}}$$

4 $${Detection}\,{percentage}( \% D)=\frac{{true}\,{positives}}{{true}\,{positives}+{false}\,{negatives}}* 100$$

5 $${Quality}\,{percentage}( \% Q)=\frac{{true}\,{positives}}{{true}\,{positives}+{false}\,{positives}+{false}\,{negatives}}* 100$$

(V) Analysis of the landscape: From the results of the supervised classification, in which forest and non-forest cover maps were obtained, indices were generated to quantify changes in the landscape configuration to measure basic characteristics: (i) Composition (number of patches—NP, largest patch index—LPI and average size of patches—AREA_MN); (ii) Shape (Average Shape Index of the patches—SHAPE_MN and average fractal dimension FRACT_MN), and (iii) Configuration (average distance to the nearest neighbor ENN_MN) of the fragments at class and landscape levels (Rutledge 2003; Vila et al. 2006). The respective calculation was carried out using the Fragstats v4.2 open software (McGarigal et al. 2012), taking forest (F) and no forest (NF) covers as classes.

Finally, the area changes of the forest cover were calculated to estimate loss or gain of this cover during the study period (Table 5).Where:Ln= Natural logarithm; A_1_ = Total surface of the cover analyzed for the initial year; A_2_ = Total surface of the cover analyzed in the final year or of change; T_1_ = Initial time and T_2_ = Final time or of change(IDEAM et al. 2002).

**Table 5:** Indices used to calculate changes in forest cover area

Index	Formula
Annual change rate (ACR)	${\boldsymbol{ACR}}=\frac{{\boldsymbol{Ln}}{{\boldsymbol{A}}}_{{\boldsymbol{2}}}-{\boldsymbol{Ln}}{{\boldsymbol{A}}}_{{\boldsymbol{1}}}}{{{\boldsymbol{T}}}_{{\boldsymbol{2}}}-{{\boldsymbol{T}}}_{{\boldsymbol{1}}}}$* 100
Change in areas (D_A_)	${{\boldsymbol{D}}}_{{\boldsymbol{A}}}={{\boldsymbol{A}}}_{{\boldsymbol{2}}}-{{\boldsymbol{A}}}_{{\boldsymbol{1}}}$
Proportion of change (PC)	${\boldsymbol{PC}}=\frac{{{\boldsymbol{A}}}_{{\boldsymbol{2}}}-{{\boldsymbol{A}}}_{{\boldsymbol{1}}}}{{{\boldsymbol{A}}}_{{\boldsymbol{1}}}}$* 100
Mean annual change (MAC)	${\boldsymbol{MAC}}=\frac{{{\boldsymbol{A}}}_{{\boldsymbol{2}}}-{{\boldsymbol{A}}}_{{\boldsymbol{1}}}}{{{\boldsymbol{T}}}_{{\boldsymbol{2}}}-{{\boldsymbol{T}}}_{{\boldsymbol{1}}}}$

## Results

Following the methodology proposed, the results obtained regarding the supervised classification and analysis of changes in the landscape’s structure and configuration for the study area during the periods analyzed are introduced hereinafter.

### Supervised Classification

Based on the results of the Kappa index obtained from the confusion matrix (Table 6), the best classifications were obtained with the CART algorithm for 2002 (July 26, dry season) and with the Random Forest algorithm for years 2014 and 2019. In 2002, the best classification was obtained from combination 4 “Spectral bands + topographic variables” with a Kappa value of 0.867; in turn, for years 2014 and 2019, the best classifications corresponded to combination 5 “Spectral indices + topographic variables”, with Kappa values of 0.839 and 0.882, respectively, with the latter being the highest value obtaictions conducted (Table 6).

These Kappa values > 0.75 evidenced a high correspondence level; added to the overall precision (OP) of the maps with values > 0.93 and the user precision (UP), as well as producer precision (PP) > 0.95 for no forest (NF) cover and >0.88 for forest (F) cover for the three best classifications (Table 6). It is worth highlighting that the CART algorithm also obtained other high Kappa index values > 0.8 in the other classifications with the six combinations for the periods studied, while the SVM and Naive Bayes algorithms had the lowest precision values, especially for combination 2 “spectral indices” (Table 6).

**Fig. 3:**
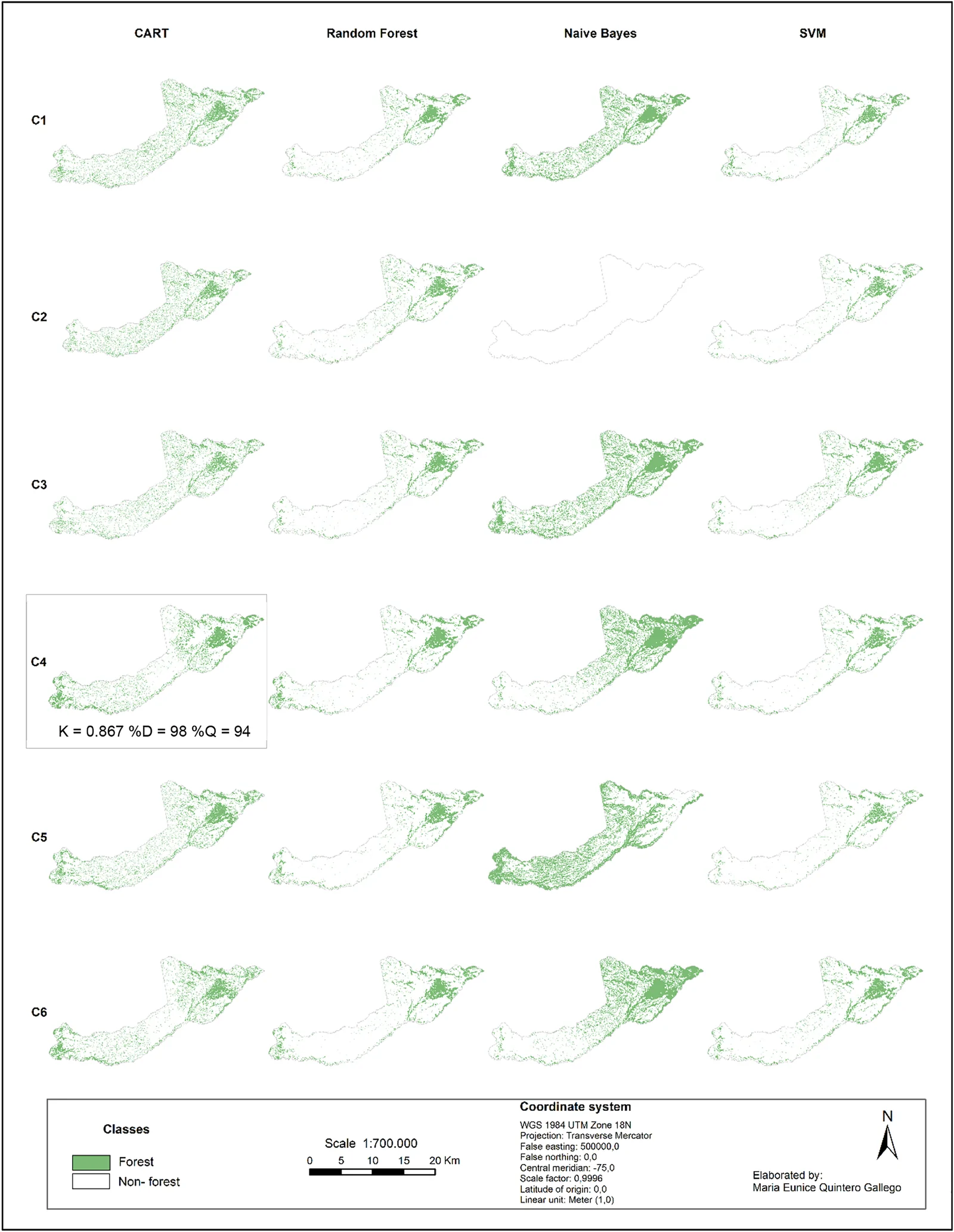
Forest classification maps for year 2002 derived from Landsat 7 ETM+ using CART, Random Forest, Naive Bayes, and SVM algorithms with six data combinations: (C1)= Spectral bands; (C2)= Spectral indices; (C3)= Spectral bands + spectral indices; (C4)= Spectral bands + topographic variables; (C5)= Spectral indices + topographic variables; and (C6)= Spectral bands + spectral indices + topographic variables

These results are reflected on the maps generated for each of the years analyzed, showing that for year 2002, the CART algorithm with combination 4 had the best classification of forest covers with the highest Kappa index, followed by combination 6 for the CART and Random Forest algorithms (Fig. 3).

**Table 6:** Overall precision, user, producer, and Kappa index measurements derived from the confusion matrix

Combination	Item	CART						Random Forest						Naive Bayes						SVM					
		2002		2014		2019		2002		2014		2019		2002		2014		2019		2002		2014		2019	
		NF	F	NF	F	NF	F	NF	F	NF	F	NF	F	NF	F	NF	F	NF	F	NF	F	NF	F	NF	F
**C1: Spectral bands**	UP	0.96	0.88	0.94	0.84	0.96	0.90	0.96	0.84	0.96	0.85	0.97	0.86	0.76	0.71	0.62	0.88	0.65	0.87	0.94	0.48	0.89	0.53	0.89	0.69
	PP	0.97	0.86	0.93	0.85	0.96	0.90	0.95	0.86	0.94	0.89	0.94	0.92	0.90	0.46	0.92	0.50	0.92	0.52	0.86	0.71	0.81	0.68	0.87	0.73
	OP	0.943		0.909		0.94		0.932		0.924		0.935		0.75		0.701		0.716		0.839		0.781		0.828	
	K	0.837		0.783		0.858		0.804		0.819		0.843		0.396		0.414		0.436		0.475		0.446		0.586	
**C2: Spectral indices**	UP	0.95	0.87	0.94	0.86	0.96	0.91	0.96	0.84	0.94	0.85	0.96	0.90	1.00	-	1.00	-	1.00	-	0.96	0.37	1.00	-	0.85	0.47
	PP	0.96	0.84	0.94	0.87	0.96	0.91	0.95	0.86	0.94	0.87	0.96	0.90	0.78	-	0.70	-	0.70	-	0.84	0.71	0.70	-	0.79	0.57
	OP	0.935		0.919		0.943		0.932		0.917		0.94		0.776		0.698		0.698		0.826		0.698		0.732	
	K	0.815		0.808		0.864		0.804		0.801		0.858		0.000		0.000		0.000		0.396		0.000		0.329	
**C3: Spectral bands + spectral indices**	UP	0.95	0.86	0.95	0.84	0.96	0.91	0.97	0.85	0.96	0.86	0.96	0.88	0.74	0.72	0.61	0.88	0.64	0.87	0.95	0.50	0.90	0.54	0.90	0.66
	PP	0.96	0.84	0.93	0.88	0.96	0.91	0.96	0.88	0.94	0.89	0.95	0.91	0.90	0.45	0.92	0.49	0.92	0.51	0.87	0.73	0.82	0.70	0.86	0.73
	OP	0.932		0.917		0.943		0.94		0.927		0.938		0.74		0.69		0.711		0.846		0.792		0.823	
	K	0.807		0.799		0.864		0.826		0.825		0.850		0.383		0.399		0.428		0.502		0.472		0.567	
**C4: Spectral bands + topographic variables**	UP	**0.96**	**0.92**	0.94	0.87	0.97	0.89	0.96	0.86	0.96	0.87	0.97	0.89	0.80	0.64	0.64	0.76	0.71	0.83	0.96	0.44	0.93	0.38	0.84	0.72
	PP	**0.98**	**0.88**	0.94	0.86	0.95	0.92	0.96	0.87	0.94	0.89	0.95	0.93	0.89	0.48	0.86	0.48	0.90	0.55	0.86	0.78	0.78	0.71	0.87	0.66
	OP	**0.953**		0.919		0.943		0.94		0.93		0.945		0.766		0.674		0.742		0.846		0.766		0.805	
	K	**0.867**		0.809		0.863		0.827		0.832		0.869		0.396		0.339		0.466		0.478		0.360		0.547	
**C5: Spectral indices + topographic variables**	UP	0.95	0.90	0.95	0.84	0.97	0.90	0.97	0.86	**0.96**	**0.88**	**0.97**	**0.91**	0.65	0.56	0.61	0.52	0.65	0.57	0.96	0.36	0.93	0.32	0.83	0.60
	PP	0.97	0.85	0.93	0.88	0.96	0.93	0.96	0.89	**0.95**	**0.89**	**0.96**	**0.93**	0.84	0.32	0.74	0.36	0.78	0.41	0.84	0.72	0.76	0.67	0.83	0.61
	OP	0.94		0.917		0.948		0.945		**0.932**		**0.951**		0.633		0.581		0.625		0.826		0.747		0.763	
	K	0.831		0.800		0.875		0.841		**0.839**		**0.882**		0.167		0.112		0.197		0.389		0.296		0.437	
**C6: Spectral bands + spectral indices + topographic variables**	UP	0.97	0.90	0.95	0.86	0.96	0.88	0.97	0.88	0.96	0.87	0.97	0.89	0.80	0.64	0.63	0.76	0.71	0.83	0.94	0.51	0.85	0.63	0.87	0.69
	PP	0.97	0.90	0.94	0.88	0.95	0.91	0.97	0.89	0.94	0.89	0.95	0.93	0,88	0,47	0,86	0,47	0,90	0,55	0,87	0,71	0,84	0,65	0,87	0,69
	OP	0.953		0.922		0.938		0.951		0.93		0.945		0.76		0.672		0.742		0.844		0.786		0.813	
	K	0.865		0.814		0.850		0.857		0.832		0.869		0.387		0.336		0.466		0.501		0.489		0.555	

UP= “User precision”PP= “Producer precision”OP= “Overall precision”K= “Kappa index”

**Fig. 4:**
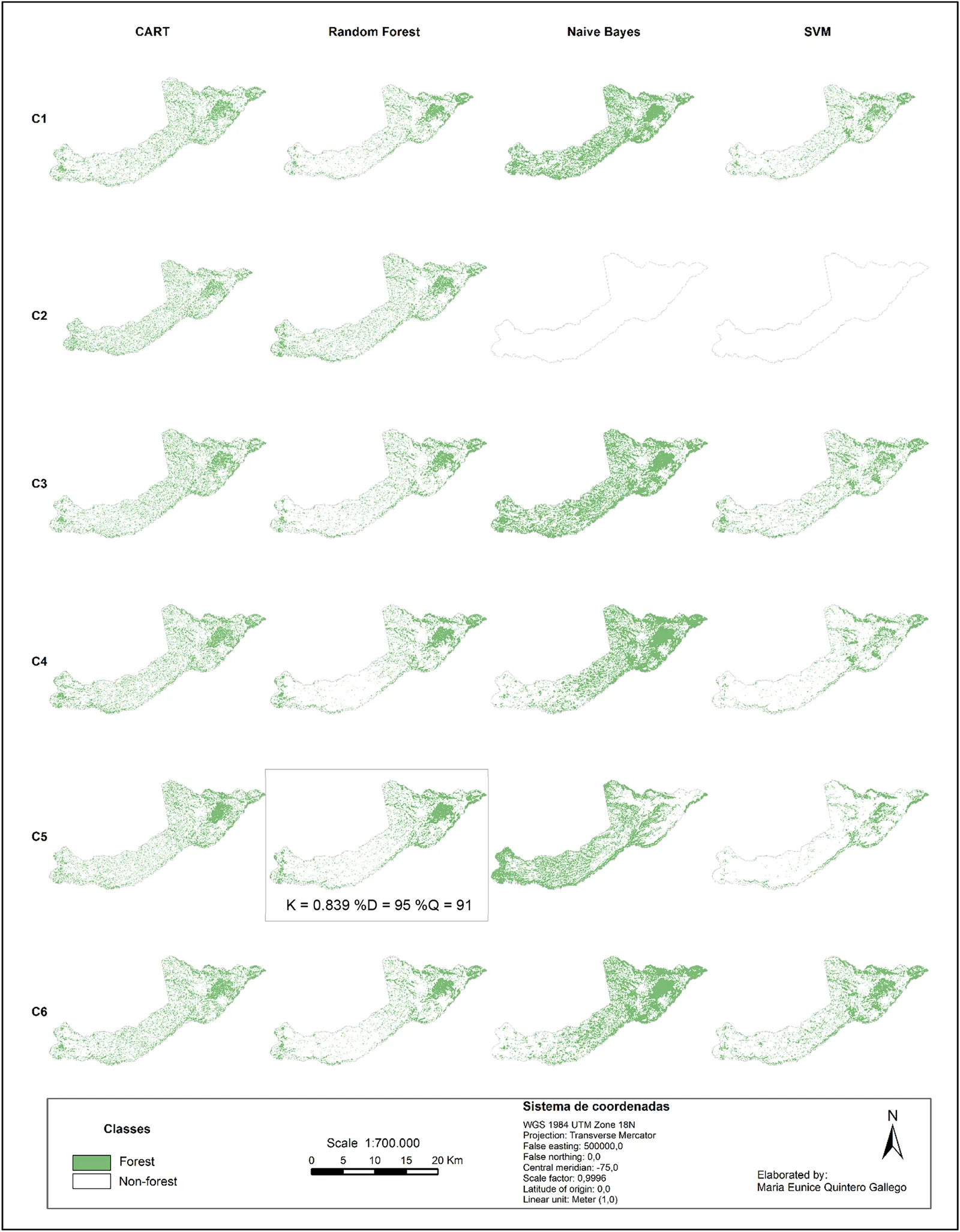
Forest classification maps for year 2014 derived from Landsat 8 OLI using CART, Random Forest, Naive Bayes, and SVM algorithms with six data combinations; : (C1)= Spectral bands; (C2)= Spectral indices; (C3)= Spectral bands + spectral indices; (C4)= Spectral bands + topographic variables; (C5)= Spectral indices + topographic variables; and (C6)= Spectral bands + spectral indices + topographic variables

**Fig. 5:**
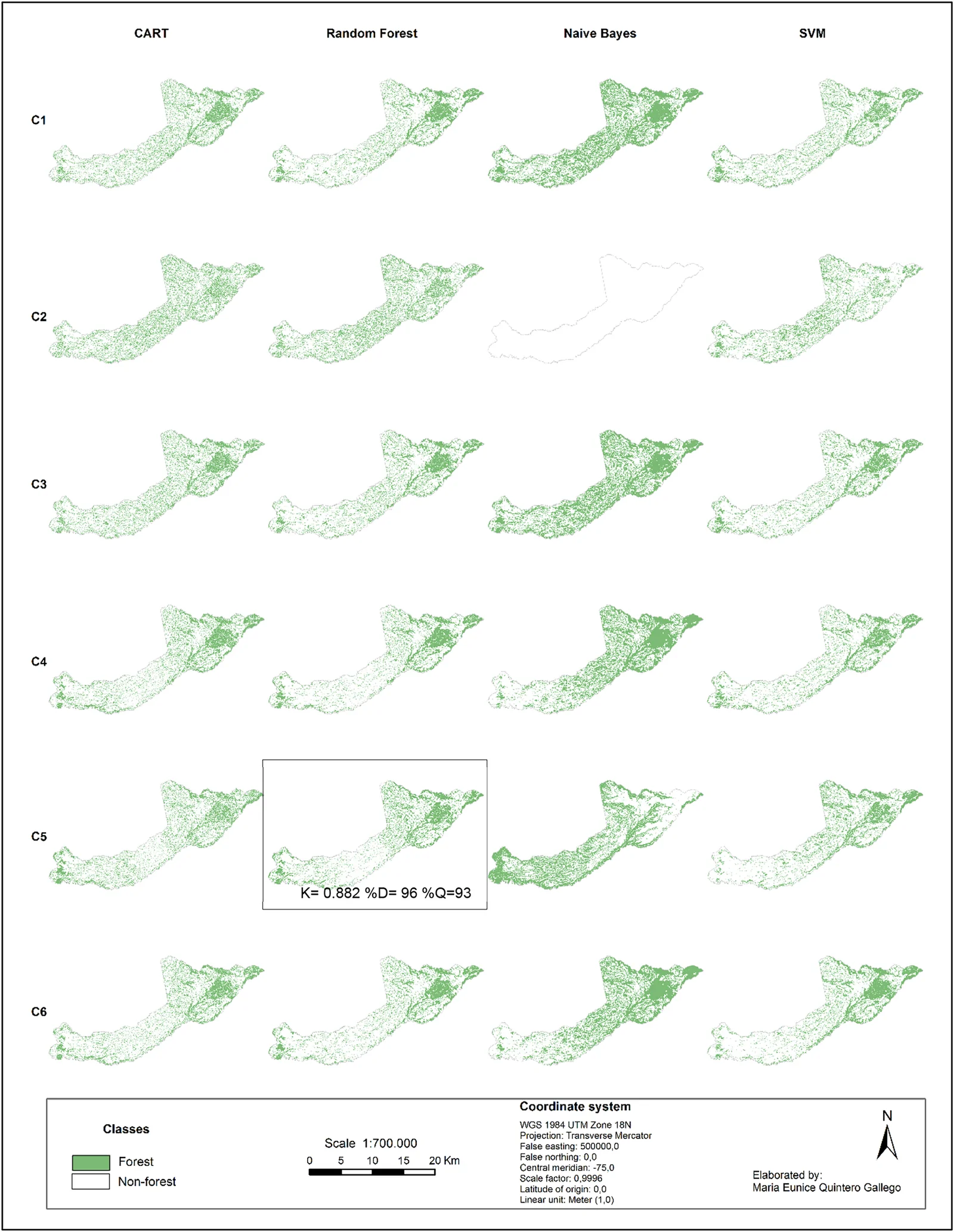
Forest classification maps for year 2019 derived from Sentinel 2 using CART, Random Forest, Naive Bayes, and SVM algorithms with six data combinations: (C1)= Spectral bands; (C2)= Spectral indices; (C3)= Spectral bands + spectral indices; (C4)= Spectral bands + topographic variables; (C5)= Spectral indices + topographic variables; and (C6)= Spectral bands + spectral indices + topographic variables

For the years 2014 and 2019, the best classification was obtained with the Random Forest algorithm combination 5 (Figs. 4, 5). For combination C2 (spectral indices only), the Naive Bayes and SVM classifiers produced classification maps in which one of the classes (Forest or Non-Forest) was nearly entirely absent, resulting in mostly black maps (Figs. 3–5).

Likewise, the classification evaluation metrics showed that the CART and Random Forest algorithms evidenced the highest precision values, with *detection percentage (%D)* > 93%, *quality percentage (%Q)* > 89%, *and branching factors (BF)* and *error factors (EF)* with values between 0.03 and 0.07, respectively, for the three study periods. The SVM and Naive Bayes algorithms had the lowest precision values with *quality percentages* between 50% and 83%, as shown in Table 7.

**Table 7:**
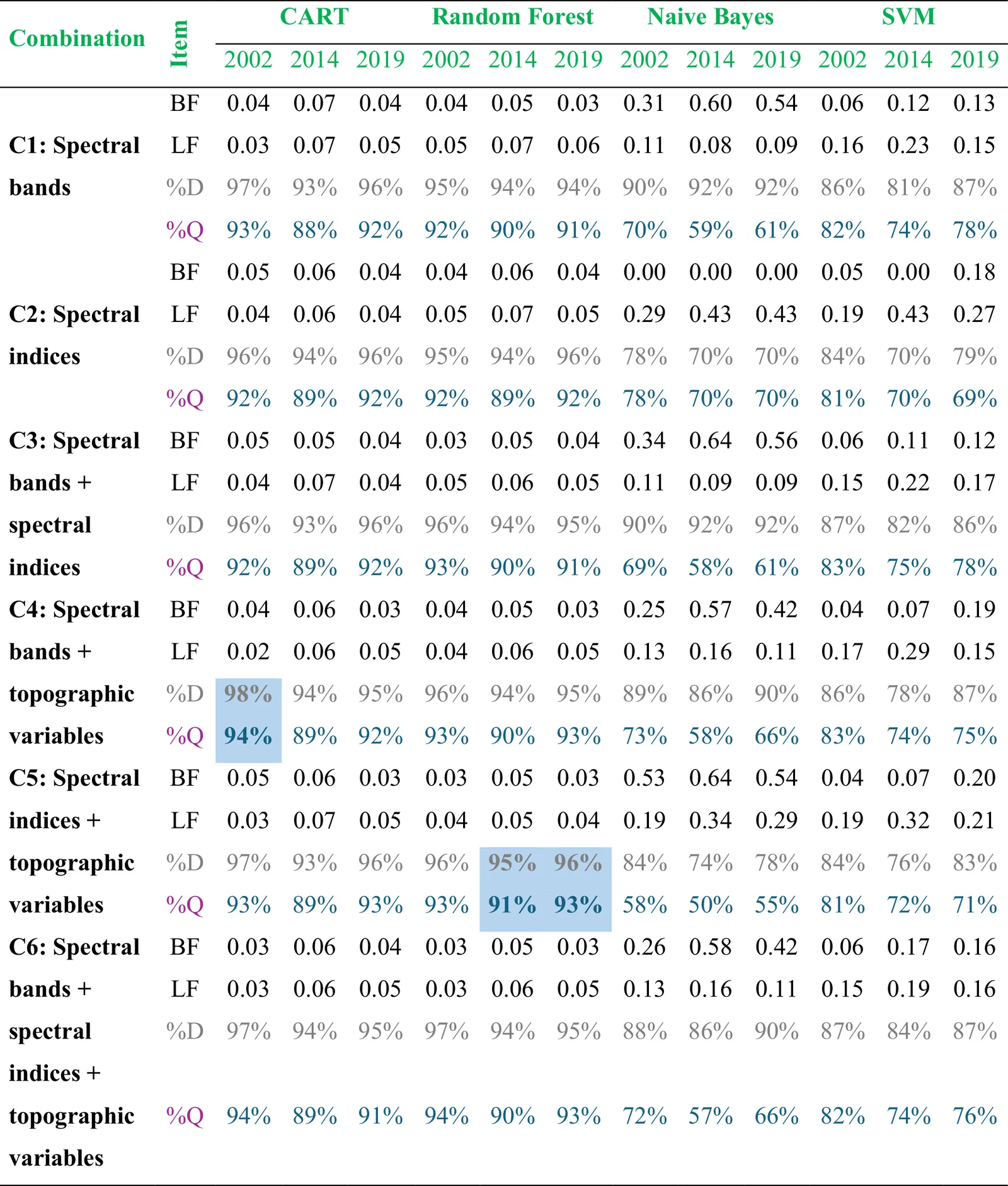
Accuracy measurements resulting from the validation confusion matrix

*BF* Branching factor, *LF* Loss factor, *%D* detection, *%%Q* quality %

### Analysis of the Landscape’s Dynamics

The landscape’s composition metrics in the study area showed that the cover of interest corresponding to forest (F) had 14.6% decrease in the number of patches (NP) from 2002 to 2014, going from 5392 to 4605. Thereafter, it had a 1.4% increase from 2014 to 2019, going to 4667 patches. The Largest Patch Index (LPI) increased during the study period, going from 1.22 in 2002 to 1.87 in 2014 and to 2.41 in 2019. The Patch Mean Size (AREA_MN) index increased from 0.79 ha in 2002 to 1.24 ha in 2019 (Table 8). The average shape index (SHAPE_MN) of the patches also increased, from 1.21 in 2002 to 1.23 in 2014 and 1.27 in 2019, indicating a change in shape. The mean fractal dimension index (FRAC_MN) remained constant at 1.04 throughout the study period (Table 8). Regarding configuration, the average distance to the nearest neighbor (ENN_MN) decreased throughout the study period, dropping from 77.42 in 2002 to 73.37 in 2019 (Table 8).

**Table 8:** Class-level indices calculated to analyze changes in the landscape’s structure and configuration

YEAR	CLASE	NP	LPI	AREA_MN	SHAPE_MN	FRAC_MN	ENN_MN
**2002**	F	5392	1.22	0.79	1.21	1.04	77.42
	NF	449	92.47	124.97	1.21	1.03	71.95
**2014**	F	4605	1.87	1.05	1.23	1.04	77.34
	NF	671	91.71	82.82	1.15	1.03	74.93
**2019**	F	4667	2.41	1.24	1.27	1.04	73.37
	NF	1092	89.92	50.11	1.17	1.03	67.97

Likewise, regarding change indicators for forest class area, an increase was observed in all four measured indicators during both periods, with the highest increase occurring during the 2014–2019 period at 19.76%, equating to an annual change rate of 3.61% (Table 9). Moreover, the analysis at the landscape level, as well as at the class level, showed a reduction in the number of patches (NP) from 2002 to 2014. The NP decreased from 5841 to 5276, which is a 9.67% reduction. Then, in 2019, there was an increase to 5759, which is a 9.2% increase. The fractal dimension index (FRACT_MN) had a value of 1.04 for all years analyzed (Table 10). These dynamics can be associated with not only fragmentation processes but also underlying deforestation or regeneration patterns. Therefore, fragmentation metrics must always be interpreted in the context of previous changes in forest cover, since loss or gain directly affects their meaning. A negative result indicates a decrease in forest area during the period analyzed. A positive result reflects an increase. A result close to zero denotes conservation or stability of the forest cover (IDEAM et al. 2002).

**Table 9:** Area change indicators, presented for the Forest class during periods 2002–2014 and 2014–2019

Indicators	Period	
	2002–2014	2014–2019
Area change (ha)	542.61	952.11
%	12.69	19.76
Mean annual change (ha/year)	45.22	190.42
Annual change rate (%)	0.99	3.61

**Table 10:** Indices at landscape level calculated to analyze changes in the landscape’s structure and configuration

YEAR	NP	FRAC_MN
**2002**	5841	1.04
**2014**	5276	1.04
**2019**	5759	1.04

## Discussion

The main contribution of the current study is its demonstration that long-term forest cover changes can be precisely tracked using an affordable approach based solely on freely accessible satellite imagery (Landsat and Sentinel) and the GEE platform. This strategy eliminates the need for costly proprietary software, advanced computational hardware, and commercial imagery, which have historically constrained systematic monitoring activities in many low- and middle-income countries (Ayyamperumal et al. 2024; Velastegui-Montoya et al. 2023). By leveraging open-access RS data with cloud-based processing power, the suggested methodology achieves high classification accuracy (overall accuracy >93%; Kappa >0.8) while significantly reducing economic and technological barriers.

The Kappa index values were 0.867 for the 2002 classification with CART, and 0.839 and 0.882 for the 2014 and 2019 classifications with Random Forest. These values indicate high classification precision, as a coefficient greater than 0.8 suggests almost complete consistency (Fu and Zhang 2022; He et al. 2022). This, combined with overall precision measurements of over 93%—which exceed the 85% threshold recommended by the US Geological Survey (USGS) for acceptable analysis of changes (Abraham and Kundapura 2022)—demonstrates that the most accurate classifications for each year were achieved using the Random Forest (RF) and CART algorithms, while the Naive Bayes and SVM algorithms produced lower precision.

Particularly, the Naive Bayes and SVM classifiers produced classification maps in which one of the classes (Forest or Non-Forest) was nearly entirely absent, resulting in mostly black maps (Figs. 3–5). This phenomenon can be explained by the inability to sufficiently separate the classes when using only spectral indices as input features. In the case of Naive Bayes, the strong independence assumption of the variables resulted in no separation, while in SVM, the reduced dimensional space of the spectral features hindered the definition of a separating hyperplane. Hence, most pixels were misclassified into a single class, as demonstrated by the Kappa statistics (0.0 for Naive Bayes and Kappa ≤0.40 for SVM) and the overall accuracy reported in Table 7. Previous studies have reported similar behavior, showing that classifiers like Naive Bayes and SVM perform poorly when only spectral indices are used, due to limited separability and high correlation between spectral bands (e.g., Grewal et al. 2023; Xie et al. 2018). These results highlight the inability of spectral features alone to distinguish between forest and non-forest areas in the study area. A more robust classification requires the inclusion of spectral bands and topographic variables.

Nevertheless, the results align with those of Talukdar et al. (2021), who achieved 81% overall accuracy and a Kappa of 0.87 when classifying a 2019 Landsat 8 image using Random Forest. Also, a study on woodland vegetation found that the CART and Random Forest algorithms achieved the greatest mapping accuracy for users (78–92%) and producers (55–77%) (Johansen et al. 2015). These results corroborate the findings of this study, in which the same algorithms achieved the highest precision values for woodland cover. The user accuracy was 92% for CART and 93% for Random Forest, and the producer accuracy was 92% for both algorithms. These precision values are critical because the binary classification of woodlands can be affected by a small sample size (Maxwell et al. 2018).

The evaluation measures validated the strength of the method. The branching factors (BF) and error factors (EF) were low, ranging from 0.03 to 0.07. The detection and quality percentages were greater than 89%. These measures are comparable to those obtained by Tapas et al. (2012) and Ariza et al. (2021) in landslide detection research. Although some studies (e.g., Basheer et al. 2022) obtained better performance using SVM, our results indicate that for this study area, Random Forest performed consistently better than SVM, which achieved only 79–85% accuracy based on the sensor. Random Forest was the most accurate classifier, as it produced the highest classifications in two out of the three periods examined. Its ensemble framework of numerous decision trees sampled independently enhances accuracy over individual-tree models (Tsai et al. 2018). In addition, Random Forest has been extensively applied in RS due to its flexibility, computational efficiency, and stability in dealing with heterogeneous variables (Belgiu and Drăgut 2016; Campos-Taberner et al. 2018).

Regarding auxiliary data, this research demonstrated that slope and elevation greatly improved classification accuracy. The highest Kappa values (0.82–0.88) were achieved when topographic features were included. These auxiliary layers help the algorithm correct for illumination effects caused by terrain and climate variation, resulting in more reliable classification (Azzari and Lobell 2017; Pimple et al. 2017). The auxiliary data is globally freely available through NASA’s SRTM, ALOS AW3D30, and Copernicus GLO-30 DEMs, as well as climate surfaces such as WorldClim and CHELSA (Farr et al. 2007; Fick and Hijmans 2017; Takaku et al. 2020). This implies that the low-cost methodological framework proposed here can be applied to a wide range of socio-ecological contexts.

Spectral indices and topographic variables improved the accuracy of the years 2014 and 2019 because vegetation indices record vigor, productivity, and moisture, enhancing discrimination between covers (Hermosilla et al. 2022). Our results are consistent with those of Pizarro et al. (2022), who showed that Random Forest performed better when using a combination of spectral and topographic variables in the Peruvian Andes. Additionally, concerning landscape metrics quantified at the class and landscape levels, the most pertinent contribution is the demonstration that a suite of biodiversity-relevant metrics can be obtained under a low-cost paradigm. Metrics such as NP, AREA_MN, LPI, and ENN_MN are widely used to measure habitat fragmentation, connectivity, and landscape integrity. These metrics are essential for conservation monitoring (Fahrig 2019; McGarigal et al. 2012; Uuemaa et al. 2009).

The findings underscore the critical need for landscape metrics that extend beyond local analyses and become an integral part of a comprehensive methodology for monitoring biodiversity. Given the central role that habitat conservation plays in protecting endangered species, methods that can automatically detect habitat loss in near real time could significantly improve compliance monitoring and enforcement capabilities, as well as substantially increase the effectiveness of conservation laws (Evans and Malcom 2021). As Turner and Gardner (2015) aptly demonstrate, landscape ecology provides a solid conceptual and practical foundation, illuminating the impact of spatial structures such as fragmentation and patch behavior on ecological processes across different spatial dimensions. Measurements such as patch numbers, mean patch dimensions, and biggest patch index are not merely descriptive; they serve as indicators of ecological soundness and robustness (Luther et al. 2020). These indicators provide valuable insights for conservation strategies and policies in various geographic settings.

For example, the findings in Quindío indicate that, at an overall level, the landscape’s dynamics are concerning. Although some indicators show an increase in the area of the fragments and a decrease in their distance, there is an increasing number of small patches, thus increasing forest fragmentation and endangering the survival of species affected by the size of the fragments (Laurance et al. 2018; Quintero et al. 2023). This is particularly challenging for species like the red howler monkey, which inhabits the Bremen and El Ocaso Mountain reserves. While some studies suggest that howler monkeys can survive in small, low-quality, and isolated fragments, these populations are not necessarily healthy or viable in the long term (Aristizábal et al. 2006). They reach a point at which the population begins to decline due to an inability to migrate to other forests. This results in decreased genetic variability, overexploitation of resources, susceptibility to epidemics, increased mortality rates, and changes in fertility rates (M. C. Gómez-Posada, 2014).

According to Gómez-Posada et al. (2006), the abundance of howler monkey populations is influenced by fragment size and area-to-perimeter ratio. However, it is not correlated with edge and isolation distance between forests because surrounding crops completely prevent dispersal of individuals and become insurmountable barriers for monkeys. This leaves few opportunities for young individuals to disperse, regardless of the distance to other forests rates (C. Gómez-Posada et al. 2006). This is evident in the endangered monkey populations located between 900 and 1200 m (“El Ocaso Mountain” natural reserve location). The monkeys live in fragmented Guadua (*Guadua angustifolia*) forests with poor plant diversity. These forests are elongated and totally isolated by crops. In contrast, populations between 1600 and 2200 m (the Bremen reserve location) inhabit forests with better environments, closer to neighboring patches, and mostly surrounded by forest plantations, which are related to the protection status of the area. These strategies are among the main tools for biodiversity conservation and generate favorable environments for the natural course of ecological processes (Chernenkova et al. 2023). Thus, studies like this become an effective low-cost tool that might support different stakeholders in the decision-making process to guide territorial ordering, public policies, and measures in favor of conserving the most vulnerable species and ecosystems.

The broader implication of the Quindío case is that these metrics can be systematically tracked using free imagery and software. This is particularly relevant for biodiversity governance in low- and middle-income countries, where limited resources frequently preclude such evaluations (Chernenkova et al. 2023; Evans and Malcom 2021). However, it is also recognized that it is critical for RS data to be used to improve conservation, that platforms like GEE continue to provide open access (Velastegui-Montoya et al. 2023). Therefore, the findings in Quindío illustrate that it is possible to combine classification accuracy assessments, auxiliary variables, and landscape metrics into a single, low-cost monitoring framework. This method has implications that extend beyond academic studies to conservation policy, ecological restoration, and land use planning. It provides decision-makers with replicable and scalable tools to monitor biodiversity-related changes on a regional scale.

## Conclusions

This study presents a cost-effective, cloud-based approach for evaluating land cover changes and their impact on biodiversity, particularly in countries with limited resources. Using Google Earth Engine, free auxiliary data, and machine learning algorithms, including Random Forest, this methodology enables rapid, large-scale studies without the need for expensive hardware or high-capacity computers. While the Quindío case study provides practical insights into landscape dynamics, its primary contribution is demonstrating that these tools can be applied to other countries using freely available data. This enables low- and middle-income countries to monitor ecosystems, inform conservation planning, and make policy decisions. Furthermore, the use of landscape metrics provides a scalable, cost-effective framework for assessing habitat structure related to biodiversity.

## Supplementary information


Supplementary information


## Data Availability

All data supporting the findings of this study are available within the paper and its Supplementary Information

## Footnotes

1 Random points analyses can be done with open-source tools like WhiteboxTools (WBT) or QGIS (https://www.whiteboxgeo.com/).
