# Supplementary material for: Low-Cost Monitoring of Spatiotemporal Changes in Forest Cover: Implications for Conservation in a Case in Quindío (Colombia)
Source: Environ Manage. 2026 Jul 17;76(8):254. doi: 10.1007/s00267-026-02491-4 (PMC13379440; doi:10.1007/s00267-026-02491-4)
Supplement: Supplementary file 1 — Supplementary information [file 267_2026_2491_MOESM1_ESM.docx]

Supplementary Material

SM 1. Data input over the study zone that was used for pre-processing and processing

| **Sensor / product** | **Spectral resolution used** | **Spatial resolution** | **Year of acquisition** | **Processing level** | **% of clouds** | **ID** |
| --- | --- | --- | --- | --- | --- | --- |
| Satellite image Sentinel 2 | “Band 2: Blue”  “Band 3: Green”  “Band 4: Red”  “Band 8: NIR”  “Band 11: SWIR 1”  “Band 12: SWIR 2” | 10 m  10 m  10 m  10 m  20 m  20 m | 2019 | Collection “COPERNICUS/S2_SR" level 2A orthorectified with surface reflectance levels or below the atmosphere (BOA). | 17.5% | ID: COPERNICUS/S2_SR/20190104T152631_20190104T153031_T18NVL |
| Satellite image Landsat 8 OLI sensor | “Band 2: Blue”  “Band 3: Green”  “Band 4: Red”  “Band 5: NIR”  “Band 6: SWIR 1”  “Band 7: SWIR 2” | 30 m  30 m  30 m  30 m  30 m  30 m | 2014 | Collection “LANDSAT/LC08/C02/T1_L2” collection 2 level 1 orthorectified with surface reflectance levels | 44.6% | ID: LANDSAT/LC08/C02/T1_L2/LC08_009057_20140719 |
|  | Band 8: Panchromatic | 15 m | 2014 | Collection “LANDSAT/LC08/C01/T1_TOA” collection 1 orthorectified with “reflectance levels above the atmosphere (TOA)” | 44.6% | ID: LANDSAT/LC08/C01/T1_TOA/LC08_009057_20140719 |
| Satellite image Landsat 7 ETM+ sensor | Band 2: Blue  Band 3: Green  Band 4: Red  Band 5: NIR  Band 6: SWIR 1  Band 7: SWIR 2 | 30 m  30 m  30 m  30 m  30 m  30 m | 2002 | Collection “LANDSAT/LE07/C02/T1_L2” level T1 orthorectified with reflectance levels below the atmosphere (BOA). | 39.0% | ID: LANDSAT/LE07/C02/T1_L2/LE07_009057_20020726 |
|  | Band 8: Panchromatic | 15 m | 2002 | Collection “LANDSAT/LE07/C01/T1_TOA” level 1 orthorectified with “reflectance levels above the atmosphere (TOA)” | 39.0% | ID: LANDSAT/LE07/C01/T1_TOA/LE07_009057_20020726 |
| DEM | N/A | 30 m | 2002 |  | N/A | ID: JAXA/ALOS/AW3D30_V1_1 |

**SM 2**. Indices and variables used for supervised classification

| **No.** | **Index/variable** | **Formula** | **Reference** | **Examples of use in remote sensing** |
| --- | --- | --- | --- | --- |
| 1 | NDVI “Normalized Difference Vegetation Index” | $NDVI= \frac{NIR-RED}{NIR+RED}$ | (Rouse Jr et al., 1974) | Classify land use and land cover (Zhao et al., 2024); (Bogale et al., 2025), Supervised classification to map flood dynamics in wetlands (Senanayake et al., 2023),  Mapping of terrestrial coverage (Pech-May et al., 2022) |
| 2 | EVI “Enhanced Vegetation Index” | $EVI= \frac{G*(NIR-RED)}{(NIR+C1) *(RED-C2)*(BLUE+L)}$  *G = 2.5*  *C1 = 6*  *C2 = 7.5*  *L = 1* | (A. Huete et al., 2002) | Supervised classification to map flood dynamics in wetlands (Senanayake et al., 2023). |
| 3 | SAVI “Soil Adjusted Vegetation Index” | $SAVI= \frac{(NIR-RED)}{(NIR+RED+L)*(1+L)}$  *L = 0.5* | (A. R. Huete, 1988) | Supervised classification to map flood dynamics in wetlands (Senanayake et al., 2023). |
| 4 | CIgreen “Chlorophyll Index Green” | $GCI= \frac{NIR}{GREEN}-1$ | (Gitelson et al., 2003) | Classification of grassland land cover (He et al., 2026) |
| 5 | NDBI “Normalized Difference Built-up Index” | $BNDI= \frac{SWIR1-NIR}{SWIR1+NIR}$ | (Zha et al., 2003) | Classify land use and land cover (Zhao et al., 2024); (Bogale et al., 2025). |
| 6 | NDWI “Normalized Difference Water Index” | $NDWI= \frac{NIR-SWIR1}{NIR+SWIR1}$ | (Gao, 1996) | Supervised classification to map flood dynamics in wetlands (Senanayake et al., 2023). |

**References**

Bogale, T., Degefa, S., Dalle, G., & Abebe, G. (2025). Machine learning-based analysis of land use and land cover trends in southeastern Ethiopia using Google Earth Engine. *Discover Sustainability*, *6*(1), 878. https://doi.org/10.1007/s43621-025-01709-5

Gao, B. (1996). NDWI—A normalized difference water index for remote sensing of vegetation liquid water from space. *Remote Sensing of Environment*, *58*(3), 257–266. https://doi.org/https://doi.org/10.1016/S0034-4257(96)00067-3

Gitelson, A. A., Gritz, Y., & Merzlyak, M. N. (2003). Relationships between leaf chlorophyll content and spectral reflectance and algorithms for non-destructive chlorophyll assessment in higher plant leaves. *Journal of Plant Physiology*, *160*(3), 271–282. https://doi.org/10.1078/0176-1617-00887

He, X., Zhang, R., Shama, A., Lv, J., Hong, R., Yang, Y., Jiang, H., Qin, J., & Liu, G. (2026). Dynamic monitoring of grassland land cover types of Inner Mongolia 1990–2023 and testing the causal relationship between meteorological data and grassland area. *Geomatics, Natural Hazards and Risk*, *17*(1). https://doi.org/10.1080/19475705.2026.2621845

Huete, A. R. (1988). A soil-adjusted vegetation index (SAVI). *Remote Sensing of Environment*, *25*(3), 295–309. https://doi.org/https://doi.org/10.1016/0034-4257(88)90106-X

Huete, A., Didan, K., Miura, T., Rodriguez, E. P., Gao, X., & Ferreira, L. G. (2002). Overview of the radiometric and biophysical performance of the MODIS vegetation indices. *Remote Sensing of Environment*, *83*(1), 195–213. https://doi.org/https://doi.org/10.1016/S0034-4257(02)00096-2

Pech-May, F., Aquino-Santos, R., Rios-Toledo, G., & Posadas-Durán, J. P. F. (2022). Mapping of Land Cover with Optical Images, Supervised Algorithms, and Google Earth Engine. *Sensors*, *22*(13), 4729. https://doi.org/10.3390/s22134729

Rouse Jr, J. W., Haas, R. H., Deering, D. W., Schell, J. A., & Harlan, J. C. (1974). Monitoring the vernal advancement and retrogradation (green wave effect) of natural vegetation. (No. E75-10354).

Senanayake, I. P., Yeo, I. Y., & Kuczera, G. A. (2023). A Random Forest-Based Multi-Index Classification (RaFMIC) Approach to Mapping Three-Decadal Inundation Dynamics in Dryland Wetlands Using Google Earth Engine. *Remote Sensing*, *15*(5). https://doi.org/10.3390/rs15051263

Zha, Y., Gao, J., & Ni, S. (2003). Use of normalized difference built-up index in automatically mapping urban areas from TM imagery. *International Journal of Remote Sensing*, *24*(3), 583–594. https://doi.org/10.1080/01431160304987

Zhao, Z., Islam, F., Waseem, L. A., Tariq, A., Nawaz, M., Islam, I. U., Bibi, T., Rehman, N. U., Ahmad, W., Aslam, R. W., Raza, D., & Hatamleh, W. A. (2024). Comparison of Three Machine Learning Algorithms Using Google Earth Engine for Land Use Land Cover Classification. *Rangeland Ecology & Management*, *92*, 129–137. https://doi.org/10.1016/j.rama.2023.10.007
